# The effects of induced hypertension on cerebral perfusion during delayed cerebral ischaemia in aneurysmal subarachnoid haemorrhage: a randomised clinical trial

**DOI:** 10.1186/2197-425X-3-S1-A815

**Published:** 2015-10-01

**Authors:** CS Gathier, JW Dankbaar, M Van der Jagt, GJE Rinkel, WM Van den Bergh, AJC Slooter

**Affiliations:** University Medical Center Utrecht, Utrecht, Netherlands; Erasmus MC University Medical Center, Rotterdam, Netherlands; University Medical Center Groningen, Groningen, Netherlands

## Introduction

Induced hypertension is often used to treat delayed cerebral ischaemia (DCI) after aneurysmal subarachnoid haemorrhage (aSAH) by trying to augment cerebral blood flow (CBF) [1]. However, evidence on effectiveness is lacking, since previous studies on the effect of induced hypertension on CBF are scarce, small and without control groups [2–4].

## Objectives

To investigate the effect of induced hypertension in treatment of DCI on CBF in a multi-centre randomised clinical trial.

## Methods

Patients with aSAH and occluded aneurysm who developed clinical signs of DCI were randomised to either induced hypertension or no induced hypertension. Hypertension was induced with norepinephrine. Cerebral perfusion was assessed with CTP as CBF (ml/100g/s) in predefined regions (ROIs) on two time points: CTP1) at time of clinical deterioration and CTP2) 24 - 36 hours after randomisation. CBF was assessed by an observer blinded for treatment allocation. We assessed the difference in overall and lowest CBF per treatment group (Wilcoxon Signed Rank test) and the change in overall CBF (delta overall CBF) between the treatment groups (Mann Whitney U test).

## Results

The MAP was 12 mmHg (95% confidence interval 8.6 - 14.5) higher in the hypertension-group (n = 12) compared to the no hypertension-group (n = 13) between CTP1 and CTP2. Delta overall CBF was 1.0 ml/100g/min (range -31 to 43) in the hypertension-group and -9.7 ml/100g/min (range -42 to 30) in the no hypertension-group (p = 0.25). In the ROI with lowest CBF at CTP1, CBF at CTP2 was significantly higher in the hypertension-group (37 [range 19 to 55] to 51 [range 16 to 79] ml/100g/s, p = 0.05), but not in the no hypertension-group (39 [range 17 to 54] to 37 [range 24 to 91] ml/100g/s, p = 0.51).

## Conclusions

The change in overall CBF did not differ between treatment groups. However, we cannot exclude that CBF improves with induced hypertension in those areas of the brain with lowest perfusion.

## Grant Acknowledgment

C.S. Gathier is supported the Dutch Heart Foundation (grant 2009B046) and the Brain Foundation Netherlands (grant 2009(1)-72).
